# Leitfaden der Hygiene

**Published:** 1897-03

**Authors:** 


**Leitfaden der Hygiene.** Von Dr. Aug. Gärtner. Zweite, vermehrte und verbesserte Auflage. Pp. xii., 407. Berlin: S. Karger. London: Williams and Norgate. 1896.

This introduction to hygiene is not intended to take the place of larger works which have been poured out in such numbers in Germany of late, and to which reference is given at the end of each chapter. It is comprehensive, well arranged, and contains a large amount of information likely to be of value for the readers for whom it is designed. The printing is admirable, and the illustrations are good and numerous. The second edition has been revised and improved in many ways, and translations into Italian and French have already proved the value of the work.

